# “Prevention of opioid use disorder: the HOME (housing, opportunities, motivation and engagement) feasibility study”

**DOI:** 10.1186/s12954-021-00560-x

**Published:** 2021-11-08

**Authors:** Kelly J. Kelleher, Ruri Famelia, Tansel Yilmazer, Allen Mallory, Jodi Ford, Laura J. Chavez, Natasha Slesnick

**Affiliations:** 1 Center for Child Health Equity and Outcomes Research, Nationwide Children’s Hospital, 700 Children’s Drive, Columbus, OH 43205 USA; 2 Department of Human Sciences, College of Education and Human Ecology, The Ohio State University, 1787 Neil Ave, 130 Campbell Hall, Columbus, OH 43201 USA; 3 College of Nursing, The Ohio State University, Newton Hall, 1585 Neil Avenue, Columbus, OH 43201 USA

**Keywords:** Housing, Youth, Homelessness, Intervention, Pilot program, Substance use risk

## Abstract

Young adults experiencing homelessness are at high risk of opioid and other substance use, poor mental health outcomes, exposure to trauma, and other risks. Providing access to stable housing has the potential to act as a powerful preventive intervention, but supportive housing programs have been studied most often among chronically homeless adults or adults with serious mental illness. The Housing First model, which does not precondition supportive housing on sobriety, may reduce drug use in homeless adults. In the present study, we piloted an adapted model of Housing First plus prevention services that was tailored to the needs of young adults (18–24 years) experiencing homelessness in the USA. Preventive services were added to the Housing First model and included youth-centered advocacy services, motivational interviewing, and HIV risk prevention services. This model was piloted in a single-arm study (*n* = 21) to assess the feasibility, acceptability, and initial efficacy of a Housing First model over a 6-month period in preparation for a larger randomized trial. We use repeated measures ANOVA to test for changes in alcohol and drug use (percent days of use; alcohol or drug use consequences), housing stability, social network support, and cognitive distortions over 6 months of follow-up. A total of 17 youth completed the study (85% retention), and a high proportion of youth were stably housed at 6-month follow-up. Participation in intervention services was high with an average of 13.57 sessions for advocacy, 1.33 for MI, and 0.76 for HIV prevention. Alcohol use did not change significantly over time. However, drug use, drug use consequences, and cognitive distortions, and the size of youths’ social networks that were drug using individuals decreased significantly. The Housing First model appeared to be feasible to deliver, and youth engaged in the supportive intervention services. The study demonstrates the potential for an adapted Housing First model to be delivered to youth experiencing homelessness and may improve outcomes, opening the way for larger randomized trials of the intervention.

## Introduction

Housing First is a model of supportive housing that provides persons experiencing homelessness with immediate access to shelter in independent living without prerequisites. Traditional housing services often require graduated access to shelter upon attainment of sobriety or acceptance of a particular volume of services. In contrast, Housing First asserts that shelter is a right and should not be contingent upon sobriety or specific services.

Originally developed by Pathways to Housing [1, 2], Housing First models to date target elderly with significant disabilities, adults with severe mental disorders and older patients with high-cost medical conditions. In general, Housing First has been effective at increasing the number of days housed for these populations, reducing costs for medical and jail services, and increasing the receipt of traditional services [3, 4]. It is less clear whether Housing First reduces substance use or improves mental health conditions over time especially for younger adults. Moreover, it has not been evaluated among youth or with regards to prevention of substance use disorder.

Gaetz [5] argues that youth are an ideal population for a modified version of Housing First. Intervening early would allow prevention services to be delivered and to house youth before they become part of the chronically homeless population since chronically homeless have extremely high rates of drug and alcohol use, premature death, and criminal justice involvement [6, 7]. For youth, persistent homelessness is associated with increased risk of human trafficking and high rates of opioid use. Gaetz also recommends modifying the Housing First model to include youth-focused case management or advocacy and diverse housing options given the many ways youth become homeless. Because of its success with adults with a variety of conditions, Housing First has been implemented with a youth focus in Northern Europe, California and several cities in the eastern and midwestern USA [8], but there are no data published on the characteristics of youth included in the model, the services they received, or the initial experience.

In response, our team conducted a prior US study [9] in which we delivered an adaptation of Housing First focused on a subsample of youth experiencing homelessness—young mothers and their babies. Specifically, women with young children (*N* = 60) were randomly assigned to either a Housing First-type model (*n* = 30) or treatment as usual (TAU) linked to a shelter (*n* = 30). Women received three months of utility and rental assistance plus an advocacy program, Strength-Based Outreach and Advocacy (SBOA), a previously trialed prevention program that relies on building self-efficacy and aiding youth in connection with community services. The housing intervention with the other prevention services yielded decreased drug use (Cohen's *d* = 0.61) and more independent living days (Cohen's *d* = 0.63) over usual shelter services. Two-thirds of women in the housing intervention were successful in maintaining their apartments six months after rental assistance ended through obtaining jobs or public assistance enrollment.

In the present study, we build on this prior work by expanding the sample to include a more diverse group of US youth experiencing homelessness and to add a longer period of rental and utilities support (up to 6 months). In addition, we further adapted the model with additional preventive services in order to explore the potential of Housing First for prevention of opioid use disorder (OUD) and related risks because up to 79% of homeless youth use opioids. To that end, the Housing First model was combined with SBOA, as well as other risk prevention services including motivational interviewing and HIV prevention, each detailed below. We present the results among the study’s sample of 21 youth from a large Midwestern city and discuss our findings as proof of concept for public housing authorities and US grant funders to expand research on this model.

## Methods

**Fig. 1:**
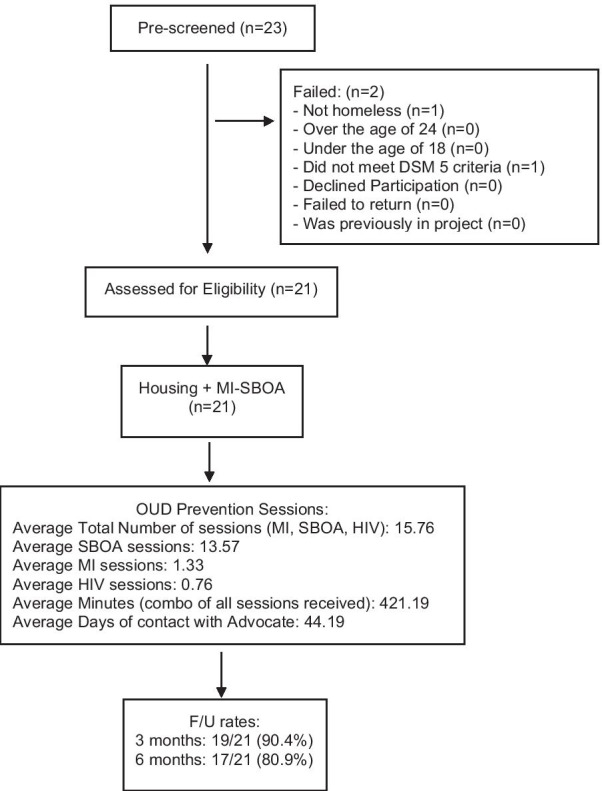
The HOME Project Phase I Consort

This was a single-arm longitudinal feasibility study of a modified Housing First model for youth. Youth were recruited from the drop-in center for homeless youth in Columbus, OH. We included youth between the ages of 18 to 24 years who were lacking a fixed, regular, stable, and adequate nighttime residence and included living in a publicly or privately operated shelter designed to provide temporary living accommodations, or a public or private place not designed for, or ordinarily used as, regular sleeping accommodations and b) did not have existing Opioid Use Disorder. The Structured Clinical Interview for DSM-5 Disorders (SCID) is a semi-structured diagnostic interview that is used to make DSM-5 diagnoses [10] for baseline eligibility. After the brief screening and stated interest in the project, written consent was obtained. Because of the potential offer for housing among those experiencing homelessness, many individuals at the drop-in center sought enrollment in the study. Because the criteria were posted, almost all were eligible to participate in the limited pilot (Fig. 1). A total of 21 youth enrolled in the study.

### Intervention: modified housing first for youth with SBOA

All youth in our study received six months of rent and utilities plus SBOA and preventive interventions focused on HIV and opioids. The focus of the study was on opioid prevention since so many youth experiencing homelessness progress to drug use on the streets and the related problems of HIV and other sexually transmitted infections.

#### Strength-based outreach advocacy

SBOA has been previously published as an effective intervention for engaging youth experiencing homelessness. It relies on unconditional acceptance, identification of specific youth skills or strengths and increasing self-efficacy. In this case, it is also focused on identifying and engaging youth from the streets and drop-ins/shelters, etc., and assisting these youth to meet their basic needs (i.e., referrals to food pantries), obtain government entitlements (i.e., cash assistance, food stamps), and connect to other needed supports (education, job training). The advocates provided referrals and/or transportation of youth to appointments as needed. Advocates were available 24 h for crises.

#### Housing

The advocate worked with the youth to identify appropriate scattered site housing among the available choices and initiated the procedure for payment directly to the landlord once housing was identified. The project covered damage deposit, application fees (including rental history, credit report), and automatic rent payments to the landlords and utility companies at the beginning of each month.

#### HIV prevention

Every youth was scheduled for a 2-session intervention which uses cognitive-behavioral techniques with a focus on skills building/behaviors (role plays with condom application, cleaning needles, communication/negotiation and problem solving [11, 12]).

#### Motivational interviewing (MI)

Motivational interviewing was added to the prevention package because it has been shown effective in reducing frequency and quantity of drug use, it is easily administered in settings on the street and in shelters in contrast to more traditional preventive interventions, and it assists homeless in prioritizing goals and efforts to help stay off the street and employed. The advocate administered MI from a MI manual adapted for homeless/runaway youth in prior trials in consultation with William R. Miller and Bo Miller (NIAAA grant no. R01AA12173 and NIDA grant R29DA11590). Adaptation of the manual included attention to the unique life situation of homeless youth in understanding motivations and challenges to recovery while homeless.

### Assessment

The baseline and follow-up assessment included self-report obtained from surveys and structured interviews. An interviewer-administered demographic/homeless experiences questionnaire assessing a set of core variables (including childhood abuse, intimate partner violence, and street victimization experiences). The primary measure of substance use quantity and frequency was assessed through the Form 90 Substance Use Interview [13]. The percentage days of substance use (alcohol, tobacco, and other drugs) as well as percentage of days of stable housing was calculated from Form 90. Form 90 assesses substance use over the past 90 days. Problem consequences were measured using the Shortened Inventory of Problems—Alcohol and Drugs [14] (SIP-AD). The Cognitive Distortions Questionnaire [15] is a 15-item measure of cognitive distortions. The Social Network Inventory (SNI) [16] has been used in multiple studies with homeless populations and high-risk adolescents [17].

### Analysis

Repeated-measures ANOVA was used to test the efficacy of the intervention (time effects). Three testing occasions (e.g., baseline, 3, and 6 months) were used as the within-subject dependent variables. The alpha level was adjusted using the Bonferroni correction. It was predicted that there would be a main effect of time; youth were expected to show reductions in opioid use and improved functioning in other domains at six months after baseline.

## Results

Attrition was the main reason for missing data in the current project. The number of participants who completed the baseline, 3-, and 6-month assessment was 21, 19, and 17, respectively. Missing data patterns were examined using Little’s MCAR test, which was not significant [*χ*^2^_(1152)_ = 10.94, *p* > 0.05] suggesting random missingness.

### Sample characteristics

Demographic characteristics are listed in Table 1. More than half of the sample (*n* = 15, 71.4%) held a job in the past 12 months. Seven youth (33.3%) reported having been arrested at least once as a juvenile, and 12 youth (57.1%) were arrested at least once as an adult (Table 2). More than half of the sample (*n* = 13, 61.9%) had received a psychiatric diagnosis from a mental health professional. Six youth (28.6%) reported suicide attempts during their lifetime. The average number of lifetime suicide attempts was 2.60, ranging from 1 to 5. The percentage of youth that reported a history of sexual, physical, and verbal abuse was 42.9% (*n* = 9), 52.4% (*n* = 11), and 57.1% (*n* = 12), respectively. During the past 12 months, youth stayed 2.86 nights on average in their own, stable housing, and their most frequently reported places of shelter were the drop-in center, followed by homeless camp.

**Table 1:** Demographic characteristics of the total sample

Variables	*n* (%)	Mean (S.D.)
Age		21.86 (1.89)
*Gender*		
Female	9 (42.9%)	
Male	11 (52.4%)	
Transitioning	1 (4.8%)	
*Ethnicity*		
African	2 (9.5%)	
Black or African American	15 (71.4%)	
Mixed	4 (19.1%)	
*Highest degree received*		
High School Diploma	11 (52.4%)	
GED	1 (4.8%)	
No Degree Received	9 (42.8%)	
*Current relationship status*		
Single, not in a relationship	14 (66.7%)	
In a relationship	7 (33.3%)	
*Number of children*		
0	16 (76.2%)	
1	3 (14.3%)	
2 and more	2 (9.5%)

**Table 2:** Baseline employment status, service needs, and housing stability

Variables	*n* (%)
Current employment status	
Work 40 + hours a week	3 (14.3%)
Work fewer than 40 h a week	5 (23.8%)
Unemployed (actively seeking work)	12 (57.1%)
Unemployed (not actively seeking work)	1 (4.8%)
Have been arrested as a juvenile	7 (33.3%)
Have been arrested as an adult	12 (57.1%)
Have received psychiatric diagnosis from a mental health professional	13 (61.9%)
Have tried to kill yourself, attempt suicide, or placed yourself in life-threatening situations	6 (28.6%)
How many times have you attempted suicide [Mean (SD)]	2.60 (1.67)
*Abuse history—as victim*	
Sexual abuse	9 (42.9%)
Physical abuse	11 (52.4%)
Verbal abuse	12 (57.1%)
*Homeless experience*	
Placed in a foster home	9 (42.9%)
Placed in a group home	5 (23.8%)
A ward of the state	6 (28.6%)
In a homeless shelter overnight	3 (14.3%)
*How many nights did you stay (in previous year) [Mean (SD)]*	
In your stable housing (paying rent)	2.86 (13.09)
With family members in their home	38.05 (59.52)
With friends in their home	27.10 (46.74)
With romantic partner in their home	2.86 (7.68)
In a shelter or mission	17.00 (50.71)
In abandoned building or as a squat	6.90 (27.21)
In jail	0.50 (2.23)
Someplace indoors, such as a bus or a train station	0.10 (0.30)
In a homeless camp	80.71 (145.06)
Someplace outdoors, such as on the street, or in a park or alley	26.00 (74.26)
In a residential treatment program	0.05 (0.22)
Drop -In Center	106.76 (134.53)
Any place hasn’t mentioned	6.89 (28.51)

The average number of advocacy, motivational interviewing, and HIV prevention sessions with advocates was 13.57 (SD = 6.38), 1.33 (SD = 0.73), 0.76 (SD = 0.99), respectively. Overall, the average number of days of contact with their advocates was 44.19 (SD = 12.94). The average of total minutes of contact was 421.19 (SD = 186.45). These sessions and interactions were performed via in-person, text messages, phone calls, and Facebook messages.

### Assessment of initial efficacy

#### Substance use

None of the youth consumed opioids during the six months of the project (Table 3). A one-way repeated measures ANOVA showed there were no significant changes in the percentage of days alcohol use from baseline, 3-m follow-up, and 6-m follow-up. One-way repeated measure ANOVAs were conducted on the percentage of tobacco use, marijuana use, cocaine use, hallucinogens use, multiple drugs use without tobacco, and multiple drugs use without tobacco and alcohol. Drug use among participants did not show statistically significant change over time, but marijuana and other drug use decreased over time. Drug use consequences showed a decrease on the Shortened Inventory of Problems (drugs and alcohol) score in the 3 month (M_SIP_ = 1.12, SD = 2.39), and 6 month (M_SIP_ = 0.50, SD = 1.51) follow-up.

**Table 3:** Assessment of initial efficacy

Variables	Baseline mean (SD)	3-m follow-up mean (SD)	6-m follow-up mean (SD)
Percent days opioid use	0.00 (0.00)	0.00 (0.00)	0.00 (0.00)
Percent days alcohol use	9.52 (18.55)	9.57 (25.28)	9.62 (23.09)
Percent days tobacco use	80.21 (29.67)	59.00 (42.77)	52.33 (46.46)
Percent days marijuana use	70.07 (38.62)	52.46 (42.79)	46.32 (43.52)
Percent days cocaine use	0.07 (0.28)	0.00 (0.00)	0.00 (0.00)
Shortened inventory of problems	5.56 (8.64)	1.12 (2.39)	0.50 (1.51) *
Percent days housed	8.06 (24.23)	56.65 (27.29)	92.88 (18.03) *
Cognitive distortions	12.81 (11.29)	8.00 (15.80)	5.06 (11.36) *
Family network size	0.52 (0.30)	0.48 (0.50)	0.55 (0.34)
Non-family network size	0.64 (0.16)	0.35 (0.46)	0.47 (0.27) *
Drug users support	0.83 (0.27)	0.25 (0.49)	0.28 (0.38) *

*Significant at *p* < .01

#### Housing

On average, in the percentage of days housed significantly increased over time (*F*_[2,30]_ = 77.18; *p* < 0.01) from 8.06% (SD = 24.23) at baseline to 92.88% (SD = 18.03) at 6-m follow-up. The increase was clearly due to the housing provided by the study. Because this pilot was preparatory for a long-term randomized trial, youth were not followed beyond six months, but advocates assisted subjects in finding jobs and enrolling in county rent supplements wherever possible.

#### Cognitive distortions

Cognitive distortions from the CD-QUEST improved over time from baseline (M_CD-QUEST_ = 12.81, SD = 11.29) to 6-m follow-up (M_CD-QUEST_ = 5.06, SD = 11.36).

#### Social network

On average, over six months of the treatment, participants reported no changes in the family network size, but non-family network size reduced significantly (*F*_[2,30]_ = 5.39; *p* = 0.01, Table 3) from baseline (M_Non-fam_ = 0.64, SD = 0.16) to 6-m follow-up (M_Non-fam_ = 0.47, SD = 0.27). Moreover, the frequency of support from drug using individuals was significantly reduced (*F*_[2,30]_ = 11.70; *p* < 0.001.). These changes might be due to supports that youth received from the advocates and a change in network characteristics as a result of housing stability.

## Discussion

Our experience with this pilot study of a youth-adapted Housing First model extends our previous study on Housing First for young mothers experiencing homelessness to a broader group and provides more detail on youth experiencing homelessness in our community. The 21 largely minority youth represented a very high-risk group with marked rates of drug use and suicidal behavior along with few prospects for housing. By providing housing, utilities, SBOA and other preventive services, our sample was highly engaged in the support services, remained almost completely housed, and showed improvements in cognitive functioning and drug-related consequences in the short term. Total drug use trended lower.

These findings are consistent with a larger literature on use of Housing First among older adults with mental illness, drug use, and severe medical comorbidities. Housing First improves housing stability, quality of life and impairments and arrests associated with drug use in many studies [18–20]. Still, there are questions about whether Housing First reduces drug use and improves core mental illness outcomes with inconsistent results among the studies showing better housing and reduced incarceration rates [21]. It is possible that the variations in outcomes may come from varied implementation of the Housing First model, the diverse samples employed, or may be the result of whether Housing First programs employed scattered site housing versus congregate housing. The latter may be associated with greater drug sharing among those housed, although this has not been tested formally.

Several limitations should be considered. This study was a small nonrandomized pilot study, testing initial efficacy, feasibility of recruitment and engagement in the housing and opioid prevention services. The youth were approached in a drop-in center in a large urban Midwestern city, representing a convenience sample. The findings might not generalize to youth in other parts of the country who do not access a drop-in center or shelter. However, we engaged a sample of Black and African American or mixed youth, nearly equal proportion of females (48%) to males, and a high proportion of sexual and gender minority youth (47%), consistent with previous samples.

In addition to testing for signals of initial efficacy of the prevention intervention on opioid use and secondary outcomes, this pilot study sought to test the feasibility of engaging and retaining youth experiencing homelessness in the intervention and housing. An important question was whether it would be possible to identify housing for youth and engage landlords to rent apartments to youth. This is a challenge given high rental costs, and because our youth often have prior criminal records, prior evictions, and poor credit history. However, we recruited our entire sample in three months and successfully found housing for all youth. Even using remote contact strategies like video, texting and telephone as COVID precautions, youth were very engaged.

## Data Availability

Data from the study will be released to the pubic after appropriate de-identification through the NIH HEAL Prevention Collaborative, coordinated by RTI, no later than one year after three-year outcome data are collected.

## Abbreviations

MI: Motivational Interviewing
OUD: Opioid Use Disorder
SBOA: Strength-Based Outreach and Advocacy
SCID: Structured Clinical Interview for DSM-5 Disorders
TAU: Treatment as Usual

## References

[1] Tsemberis S, Gulcur L, Nakae M (2004). Housing First, consumer choice, and harm reduction for homeless individuals with a dual diagnosis. Am J Public Health.

[2] Tsemberis S, Asmussen S (1999). From streets to homes. Alcohol Treat Q.

[3] Stergiopoulos V, Gozdzik A, Misir V (2016). The effectiveness of a Housing First adaptation for ethnic minority groups: findings of a pragmatic randomized controlled trial. BMC Public Health.

[4] Kerman N, Aubry T, Adair CE (2020). Effectiveness of housing first for homeless adults with mental illness who frequently use emergency departments in a multisite randomized controlled trial. Adm Policy Ment Health.

[5] Stephen G (2014). Can housing first work for youth?. Eur J Homeless.

[6] Coumans M, Spreen M (2003). Drug use and the role of homelessness in the process of marginalization. Subst Use Misuse.

[7] Linton SL, Celentano DD, Kirk GD, Mehta SH (2013). The longitudinal association between homelessness, injection drug use, and injection-related risk behavior among persons with a history of injection drug use in Baltimore, MD. Drug Alcohol Depend.

[8] Gaetz S. This is Housing First for Youth: A Program Model Guide. In. Toronto: Canadian Observatory on Homelessness Press. Toronto. 2017.

[9] Guo X, Slesnick N, Feng X (2016). Housing and support services with homeless mothers: benefits to the mother and her children. Community Ment Health J.

[10] First MBWJBW, Karg RS, Spitzer RL. User's Guide for the Structured Clinical Interview for DSM-5® Disorders—Research Version. Arlilngton, VA: American Psychiatric Association Publishing; 2015.

[11] Carmona J, Slesnick N, Guo X, Letcher A (2014). Reducing high risk behaviors among street living youth: outcomes of an integrated prevention intervention. Child Youth Serv Rev.

[12] Slesnick N, Kang MJ (2008). The impact of an integrated treatment on HIV risk behavior among homeless youth: a randomized controlled trial. J Behav Med.

[13] Miller WR. Form 90 a structured assessment interview for drinking and related problem behaviors. Project MATCH Monograph Series (Vol. 5). In: Vol 5. Bethesda, MD: U.S. Department of Health; 1996.

[14] Gillespie W, Holt JL, Blackwell RL (2007). Measuring outcomes of alcohol, marijuana, and cocaine use among college students: a preliminary test of the shortened inventory of problems—alcohol and drugs (SIP-AD). J Drug Issues.

[15] de Oliveira IR, Seixas C, Osório FL (2015). Evaluation of the psychometric properties of the cognitive distortions questionnaire (CD-Quest) in a sample of undergraduate students. Innov Clin Neurosci.

[16] Stein CH, Rappaport J, Seidman E (1995). Assessing the social networks of people with psychiatric disability from multiple perspectives. Community Ment Health J.

[17] Urberg K, Goldstein MS, Toro PA (2005). Supportive relationships as a moderator of the effects of parent and peer drinking on adolescent drinking. J Res Adolesc.

[18] Kirst M, Zerger S, Misir V, Hwang S, Stergiopoulos V (2015). The impact of a Housing First randomized controlled trial on substance use problems among homeless individuals with mental illness. Drug Alcohol Depend.

[19] Padgett DK, Gulcur L, Tsemberis S (2006). Housing first services for people who are homeless with co-occurring serious mental illness and substance abuse. Res Soc Work Pract.

[20] Homelessness EOo. The regulation and quality of homelessness services. In. Revised Edition ed 2019.

[21] Wang JZ, Mott S, Magwood O (2019). The impact of interventions for youth experiencing homelessness on housing, mental health, substance use, and family cohesion: a systematic review. BMC Public Health.

